# Gossypol Toxicity from Cottonseed Products

**DOI:** 10.1155/2014/231635

**Published:** 2014-05-06

**Authors:** Ivana Cristina N. Gadelha, Nayanna Brunna S. Fonseca, Silvia Catarina S. Oloris, Marília M. Melo, Benito Soto-Blanco

**Affiliations:** ^1^Programa de Pós-graduação em Ciência Animal, Universidade Federal Rural do Semi-Árido, BR 110 Km 47, 59628-360 Mossoró, RN, Brazil; ^2^Fundação Ezequiel Dias (FUNED), Rua Conde Pereira Carneiro 80, 30510-010 Belo Horizonte, MG, Brazil; ^3^Departamento de Clínica e Cirurgia Veterinárias, Escola de Veterinária, Universidade Federal de Minas Gerais, Avenida Antônio Carlos 6627, 30123-970 Belo Horizonte, MG, Brazil

## Abstract

Gossypol is a phenolic compound produced by pigment glands in cotton stems, leaves, seeds, and flower buds (*Gossypium* spp.). Cottonseed meal is a by-product of cotton that is used for animal feeding because it is rich in oil and proteins. However, gossypol toxicity limits cottonseed use in animal feed. High concentrations of free gossypol may be responsible for acute clinical signs of gossypol poisoning which include respiratory distress, impaired body weight gain, anorexia, weakness, apathy, and death after several days. However, the most common toxic effects is the impairment of male and female reproduction. Another important toxic effect of gossypol is its interference with immune function, reducing an animal's resistance to infections and impairing the efficiency of vaccines. Preventive procedures to limit gossypol toxicity involve treatment of the cottonseed product to reduce the concentration of free gossypol with the most common treatment being exposure to heat. However, free gossypol can be released from the bound form during digestion. Agronomic selection has produced cotton varieties devoid of glands producing gossypol, but these varieties are not normally grown because they are less productive and are more vulnerable to attacks by insects.

## 1. Introduction

Cotton (*Gossypium* spp.) is an arborous plant from the Malvaceae family. It is one of the earliest plants that were cultivated by man and it has been used for over 4,000 years. It is primarily cultivated for fiber used in the textile industry and the oil from the cotton seed [1]. The genus* Gossypium* spp. includes many species distributed throughout the world, but only four species are grown for cotton fiber:* Gossypium hirsutum* L.,* Gossypium barbadense* L.,* Gossypium arboreum* L., and* Gossypium herbaceum* L. The most economically important cotton species is* G. hirsutum*, which is grown to produce 90% of the world's cotton [2]. Cotton fiber and oil production generate byproducts rich in fat from oil and protein which are used for animal feeding. However, this plant contains a toxic compound, gossypol [1].

## 2. Chemistry of Gossypol

**Figure 1:**
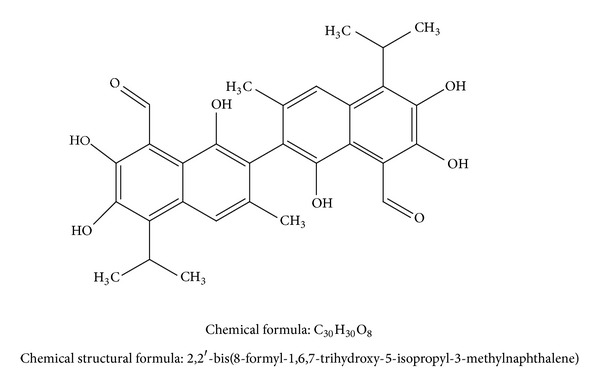
Chemical structure, formula, and structural formula of gossypol.

Gossypol is a phenolic compound that was first isolated in 1899. The name is derived from the plant genus scientific name (*Gossypium*) combined with the ending “ol” from phenol [1]. Gossypol has a 518.55 Dalton molecular weight, has a yellow pigment, is crystalline, is insoluble in water and hexane, is soluble in acetone, chloroform, ether, and methyl ethyl ketone (butanone), and is partly soluble in crude vegetable oils. The chemical formula is C_30_H_30_O_8_, and the chemical structural formula is 2,2′-bis(8-formyl-1,6,7-trihydroxy-5-isopropyl-3-methylnaphthalene) (Figure 1) [1, 3, 4].

Gossypol is produced by pigment glands in cotton stems, leaves, seeds, and flower buds. The pigment glands are small black spots distributed throughout the cotton plant but their greatest concentration is in the seeds [1, 4–6]. The seed of* G*.* barbadense* may contain up to 34 g of gossypol/kg [7]. Gossypol promotes several toxic effects in vertebrates but provides the cotton plant with resistance to pests [1, 4–6]. The pigment glands produce additional phenolic pigments (at least 14), but they are at concentrations well below the concentration of gossypol and thus have little toxicological significance [1].

Gossypol is a mixture of two enantiomers, (−) and (+) gossypol [1, 8–11]. The (−) gossypol enantiomer is more slowly eliminated [12], although it is the most biologically active form. Consequently, it is more toxic than the (+) gossypol [11, 13]. The* Gossypium* species produces both enantiomers in varying proportions, which is genetically determined [1, 9, 10, 14]. For example, the (−) gossypol proportion ranges from 33.8 to 47.0% in the seeds of upland variety (*G*.* hirsutum*) [15, 16] and from 24.9 to 68.9% in the seeds of* G*.* barbadense* [7].

Two gossypol forms have been observed, free and bound [6]. The bound form is produced via covalent bonds between gossypol and the free epsilon-amino groups from lysine and arginine [1, 17, 18] through the browning or Maillard reaction [1]. However, this reaction reduces the availability of amino acids for absorption by the animal with lysine being the most affected [18].

Total gossypol production is influenced by several factors, including weather conditions and cotton species. Considering weather conditions, gossypol production is positively correlated with the rainfall rate and negatively correlated with temperature [19]. Regarding variation among cotton species,* G. barbadense* has higher gossypol concentrations than* G. hirsutum*. On the other hand, cotton storage slightly decreases the free gossypol content [1].

## 3. Gossypol in Cotton Products

The free gossypol content in whole cotton seeds varies among the many cotton varieties [6, 20]; gossypol concentrations range from 0.02 to 6.64% [21]. Cottonseed may contain concentrations greater than 14,000 mg/kg of total gossypol and 7,000 mg/kg of free gossypol [6]. However, after oil extraction from the seeds, up to 0.6% is available following solvent extraction, but approximately 0.06% is available, if the extraction process involves mechanical pressure and heat treatment [22].

In addition to its harmful effects, gossypol and its derivatives have potential therapeutic use. These compounds showed* in vitro* action against some viruses such as human immunodeficiency virus [23, 24] and H5N1 influenza virus [24, 25] and several bacteria and yeasts [26–29]. Gossypol is a promising treatment for leukemia [30], lymphoma [31], colon carcinoma [32], breast cancer [33, 34], myoma [35], prostate cancer [36], and other malignancies [37–43]. Furthermore, it was used in China, in 1970, to treat uterine fibroids, endometriosis, and uterine bleeding in women [35].

## 4. Toxicokinetics

The gossypol absorption rate is inversely proportional to the amount of iron in the diet [71], and dietary supplementation with ferrous sulfate inactivates free gossypol [72]. In ruminants, microbial fermentation in the rumen binds dietary free gossypol with proteins [73], but it is not known whether the bound form can be absorbed by the intestines or the microorganisms can release free gossypol from the bound form. The absorbed gossypol accumulates in the liver [74] and kidneys [75]. The primary gossypol excretion route is through bile; it is then eliminated through feces after conjugation with glucuronides and sulfates [76]. In rats dosed orally with 5 mg of both racemic forms of gossypol, 70.4% of (+) and 80.2% of (−) gossypol were excreted in the feces within five days, whereas 2.30% of (+) and 2.79% of (−) gossypol were excreted in the urine [77]. Small amounts of gossypol are also excreted in expired air [1]. Little to no gossypol is excreted in the milk [74]. The half-lives (*t*_1/2_) of total (+) and (−) gossypol in rats following a single intravenous dose were estimated as 25.26 hours and 10.53 hours, respectively [77].

## 5. Gossypol Poisoning

Cottonseed includes sufficiently high gossypol concentrations to produce acute poisoning. However, there are cumulative effects of dietary gossypol and toxicity which can occur following an ingestion period of one to three months [1, 78–81]. Gossypol poisoning has been reported in many species, including broiler chicks [82], pigs [71], dogs [83, 84], sheep [85], and goats [86]. Monogastric animals, such as pigs, birds, fish, and rodents, are more susceptible to gossypol toxicity than ruminants [5, 6, 20, 87]. Moreover, young ruminants are more sensitive to gossypol compared with adult ruminants [1] because gossypol is not bound during ruminal fermentation, as it occurs in animals with fully functional rumens. However, if the gossypol intake overwhelms the ruminal detoxification capacity, free gossypol may be absorbed at hazardous concentrations even in adult ruminant animals [88].

General signs of acute toxicity are similar among animal species and include respiratory distress, impaired body weight gain, anorexia, weakness, apathy, and death after several days [1, 6, 80, 85, 89–93]. Heart failure was reported in calves [90, 94], lambs [85], and dogs [79].

The postmortem findings in ruminants include pulmonary edema, yellowish liquid in the chest and peritoneal cavities, gastroenteritis, centrilobular liver necrosis, and hypertrophic cardiac fiber degeneration. In calves, the major pathologic findings are ascites, visceral edema, acute centrilobular hepatocyte necrosis, kidney damage, and cardiovascular lesions. Increased pneumonia has also been observed, likely due to an increased sensitivity to secondary infections [85, 90–92].

Pigs may present reduced weight gain, anorexia, respiratory distress, cardiac insufficiency, coughing, and exercise intolerance. Necropsy findings include fluid accumulation in the body cavities; edema and congestion in the liver, lung, and spleen; and cardiac hypertrophy with degenerated muscle fiber [71].

Anemia is often observed in animals fed cottonseed. In fact, gossypol is a highly reactive compound that readily binds to minerals and amino acids. Binding with iron forms a gossypol-iron complex, which inhibits the absorption of this metal. The consequent iron deficiency affects erythropoiesis. Furthermore, gossypol promotes increased erythrocyte fragility [57, 74, 87, 95]. Gossypol also stimulates the eryptosis (apoptosis-like erythrocyte death) by increasing cytosolic Ca^2+^ activity resulting in cell membrane scrambling and contraction, which contributes to anemia [96].

Gossypol also affects thyroidal metabolism [68, 97–100]. Some studies with male [98] and female [99] rats showed decreased blood concentrations of T4 and T3 after dosing with gossypol. On the other hand, gossypol dosing resulted in increased T3 serum concentrations without affecting T4 in rats [97] and sheep [68]. The histopathological evaluation of thyroid glands from male rats dosed with gossypol revealed follicular degeneration and atrophy [98]. The thyrotropic cells in the pituitary gland, which are specialized for TSH synthesis and secretion, showed hypertrophy, hyperplasia, and degranulation after gossypol dosing in rats [100].

Certain clinical signs of gossypol poisoning have been attributed to reduced antioxidants in tissues and increased reactive oxygen species formation, which produces lipid peroxidation [101–104]. At high concentrations, gossypol also impairs energy generation from oxidative metabolism by interfering with enzymatic activity in the mitochondrial electron transport chain and oxidative phosphorylation [105–107]. Furthermore, gossypol decreases the contraction force of the heart and the extent of contraction of cardiac fibers [108].

## 6. Liver Damage

In addition to such effects, gossypol is hepatotoxic (Table 1) [11, 44–47, 71, 109, 110]. Ascites and hepatocyte degeneration (strong cytoplasmic eosinophilia and nuclear pyknosis) were observed in rats that received a single intraperitoneal gossypol dose of 25 mg/kg BW [45] or 30 mg/kg BW [46]. Rats that received lower gossypol doses (15 mg/kg/day for four weeks or 30 mg/kg/day for two weeks) showed morphological changes in the liver, as observed through electron microscopy, which were characterized by mitochondrial vacuolation, an enlarged endoplasmatic reticulum, an expanded perinuclear space, and collagen fiber proliferation in the perisinusoidal space [109]. Chickens fed a diet with 0.1% free gossypol for 21 days had increased plasma gamma glutamyltransferase activity and liver lipidosis [44]. Broilers that received a diet with 0.4% total gossypol for 20 days had greater liver weights [11].

## 7. Reproductive Effects

Gossypol affects male and female gametogenesis and promotes embryo lesions [81]. In the 1950s, China underwent a sharp drop in the birthrate in many rural areas where humans were consuming cottonseed oil containing gossypol. This observation was initially associated with male infertility caused by gossypol in the cottonseed oil that they were consuming. Gossypol has been investigated for use as a male contraceptive in a number of experimental studies [1, 81, 111–115].

The gossypol toxicity for male reproduction (Table 2) was reported in several studies showing that it inhibits spermatogenesis, which decreases the sperm count and spermatozoid motility and viability [20, 47–51, 53, 55, 102, 116–120]. The male antifertility effect is dose and time dependent; in effective doses, gossypol causes infertility by inhibiting sperm motility, decreasing sperm concentrations, inducing specific mitochondrial injury to the sperm tail, and damaging the germinal epithelium [20]. However, such effects are reversible when gossypol is no longer ingested [52]. Furthermore, gossypol administration to male rats did not interfere in the embryonic and fetal development of untreated dam offspring [121].

The deleterious effects on male reproduction have not been observed for all animals fed cottonseed meal. In adult male goats [122] and sheep [123] fed a diet with 0.5 kg/animal/day cottonseed meal for 120 consecutive days, no detrimental effects on semen volume, sperm concentration, motility, and morphology.

The gossypol-mediated spermatozoid disturbance mechanism includes the inhibition of release and utilization of ATP by the sperm cells [124]. Another effect of gossypol is the reduction of cellular and microtubular *β*-tubular content in spermatocytes and spermatids [125]. Furthermore, gossypol inhibits calcium influx [126, 127] and Mg-ATPase and Ca-Mg-ATPase activity in spermatozoid plasmatic membranes [126]. Abnormal spermatozoids are produced because gossypol produces ultrastructural alterations in the nuclear membrane, endoplasmic reticulum, and mitochondria [119, 128–130]. In cultivated Sertoli cells from piglets, gossypol also decreases cellular oxidase activity and damages the DNA [131]. Reduced nuclear expression of androgen receptors was observed in Leydig cells, Sertoli cells, and myoid cells from rats fed gossypol-rich cottonseed flour [132].

Gossypol also affects female reproduction (Table 3), and ruminant females tolerate higher dietary gossypol concentrations than nonruminant females [20, 54, 118, 133], probably due to the ruminal detoxification. Female exposure to gossypol has been associated with interference with the estrous cycle, pregnancy, and early embryonic development [20, 57, 81]. Gossypol interfered with rodent estrous cycles [54, 134] and pig granulosa cell function [135]. Furthermore, ovaries from heifers fed cottonseed meal had fewer large follicles (>5 mm) than heifers fed soybean meal [57]. Gossypol affected* in vitro* ovarian steroidogenesis [136, 137] as well as bovine oocyte cumulus expansion and nuclear maturation [137].

Previous studies have shown that gossypol interferes with embryonic development [118, 138–141]. In fact, gossypol may reach the uterine fluids through the maternal circulation [141]. A gossypol-mediated embryotoxic effect has been observed in* in vitro* [118, 138, 140–142] and* in vivo* [57, 139, 141, 143] studies. The early pregnancy loss promoted by gossypol is not due exclusively to direct damage to embryos but also to interference with implantation of the embryo [139]. However, this compound significantly reduced the fetal body weight in pregnant mice, but no fetal abnormalities were observed [144].

The probable mechanism for gossypol embryotoxicity is through direct embryonic cytotoxicity [20, 143]. This cytotoxic effect might be promoted by (1) generation of reactive oxygen species inducing oxidative stress [102, 104, 145], (2) intercellular communication disruption [146], (3) apoptosis induction [32, 147–152], or (4) interference with ionic transport in membranes, which increases intracellular calcium [153].

## 8. Immunotoxicity

Gossypol may cause a reduced number of leukocytes and primarily lymphocytes, which affects the immunocompetence of the organism [154].* In vivo* and* in vitro* mouse experiments also demonstrated that gossypol has immunosuppressive activity [155], which operates by affecting lymphocytes through inhibiting proliferation and inducing apoptosis [155, 156]. Mice that received gossypol had significantly decreased numbers of lymphocytes in the thymus and mesenteric lymph nodes [157], in the total spleen cell population [144], and in the capacity of blood and lymphatic cells to produce antibodies after sheep erythrocyte immunization [144, 157]. Furthermore, the spleen and lymph nodes from mice receiving gossypol had decreased CD4+ thymocyte populations and increased CD8+ lymphocyte populations [157].

The interference of gossypol with lymphocytes influence immune function as observed in a number of studies [157–160]. After inoculation with* Brucella abortus* smooth strain 99 (S99), specific anti-*Brucella *antibody production was impaired in lambs [159] and calves [160] fed cottonseed meal. Mice treated with gossypol had decreased IgM and IgG production after sheep erythrocyte immunization [157]. Men treated with gossypol as a male contraceptive showed reduced IgG titers which could be associated with altered lymphocytes [158].

*In vitro* murine macrophage proliferation was inhibited by gossypol [157]. Furthermore, rat peritoneal macrophages incubated with gossypol inhibited arachidonic acid metabolism and prostaglandin E_2_ production [161]. On the other hand, macrophage chemotaxis induced by* Edwardsiella ictaluri* challenge was increased in channel catfish (*Ictalurus punctatus*) fed cottonseed [162] or receiving gossypol [163], but catfish were unaffected by gossypol in another study [27]. Gossypol also increased serum lysozyme activity in channel catfish following an* E. ictaluri* challenge [27, 163].

## 9. Preventive Procedures

The preventive procedures at this time involve the treatment of cottonseed products to decrease the concentrations of free gossypol through the use of heat and pressure in the processing of these products (Table 4). Agronomic selection has produced cotton varieties devoid of glands producing gossypol [164], but these varieties are less grown because they are not as productive and are more vulnerable to attacks by insects [1]. One alternative is the selection and use of cotton varieties containing a relatively high (+) to (−) gossypol enantiomer ratio [13]. The directive 2002/32 of the European Union (2002L0032 - EN - 26.02.2013 - 017.001) states that the maximum free gossypol concentrations for cottonseed are 5,000 ppm and 1,200 ppm for cottonseed meal or cake and, for complete feeding stuffs, are 20 ppm for laying hens and piglets, 60 ppm for rabbits and pigs, 100 ppm for poultry and calves, and 500 ppm for cattle, sheep, and goats.

Processing including heat treatment [58, 165] and extrusion process [59] can reduce free gossypol concentrations in cottonseed. However, it is possible that the conjugate formed can release free gossypol during digestion. In fact, cows fed diets containing whole cottonseed with similar total gossypol concentrations but different free gossypol concentrations had similar total plasma gossypol [59]. Furthermore, even though the extrusion process reduced free gossypol concentration but not the total gossypol concentration; broiler chicks fed extruded cottonseed meal or feed-grade cottonseed meal showed decreased body weight gain, increased feed intake, and inefficient feed conversion rate [166].

Radiation treatment using gamma [60, 167, 168] or electron beam irradiation [60, 61] may reduce free gossypol concentrations. In fact, gossypol irradiation reduced* in vitro* prooxidative activity and embryotoxicity in mice [168]. The mechanism for gossypol destruction through radiation is unknown, but it has been speculated that gossypol molecule aggregation, gossypol cross-linking with other molecules, and gossypol molecule fragmentation or breakdown may produce such destruction [61]. On the other hand, ammoniation, which is a procedure that is used to reduce aflatoxin content of food, increased cottonseed meal toxicity in dairy cattle [169].

Some fungus may reduce free gossypol concentrations in cottonseed meal by fermentation, including* Aspergillus niger* [63, 64, 170],* Aspergillus oryzae* [62],* Candida tropicalis* [63–66],* Saccharomyces cerevisiae* [63, 64], and* Geotrichum candidum* [67]. The use of fermented cottonseed meal to feed animals seems to be safe [62, 171]. However, while these microorganisms could be used to reduce free gossypol concentration in cottonseed meal, they are not currently commercially available.

Supplementation with ferric sulfate reduces free gossypol concentrations in food due to ferric sulfate binding with reactive groups from gossypol, which forms a conjugate. The recommendation for supplementation is 1 mol of gossypol for each mol of iron, which could increase the maximum concentration of gossypol from 50 to 150 ppm for laying birds and from 100 ppm to 400 ppm for pigs and poultry [1]. Additional nutrients may be used for dietary supplementation to reduce gossypol availability. Supplementing the diet with 1 mg of sodium selenite per day in adult sheep reduced the gossypol toxicity affecting semen quality [68]. Dietary vitamin E supplementation at 4000 IU/bull/day also reversed the negative effects of gossypol on sperm production and semen quality of bulls [69] and reversed the increased erythrocyte osmotic fragility in heifers [70] promoted by feeding cottonseed meal.

Gossypol was produced as a conjugate with bovine serum albumin for vaccines. This conjugate induces antibody production against gossypol in rats, but the immunized animals were more sensitive to the acute hepatotoxic effect of gossypol [46].

## 10. Conclusions and Future Research Directions

The ingestion of gossypol present in cottonseed and its products (cakes and meal) may promote clinical poisoning, liver damage, male and female reproductive toxicity, and immunological impairment. The acute poisoning is not currently a significant problem but the reproductive damage causes serious economic losses to the livestock industry. Even though the male reproductive toxicity is well known, there is a need for more studies to understand the female reproductive damage promoted by gossypol. The immunotoxicity of gossypol is far from being completely elucidated, but it impacts animals by reducing their resistance to infections and by impairing the efficiency of vaccines. Extensive research is needed to develop more efficient and inexpensive technologies to reduce gossypol toxicity.

## Figures and Tables

**Table 1:** Experimental studies showing liver damage induced by gossypol.

Animals	Gossypol dose	Route of administration	Duration of treatment	Reference
Broiler	0.4% of total gossypol in food	Oral	20 days	[11]
Chickens	0.1% of free gossypol in food	Oral	21 days	[44]
Rats	25 mg/kg BW	Intraperitoneal	Single dose	[45]
Rats	30 mg/kg BW	Intraperitoneal	Single dose	[46]
Rats	5, 10 and 20 mg/kg BW	Intraperitoneal	10 days	[47]

**Table 2:** Selected experimental studies describing effects of gossypol on male reproduction.

Animals	Gossypol dose	Effects	Reference
Hamsters	10 mg/kg BW/day	Degeneration of spermatocytes	[48]
Rats	20 mg/kg BW/day	Degeneration of spermatocytes	[48]
Mice	40 mg/kg BW/day	No degeneration	[48]
Rats	25 mg/kg BW/day	Decreased spermatogenesis, Sertoli cell, and seminiferous tubules damage	[49]
Rats	10 mg/kg BW/day	Tubular degeneration, reduced testosterone concentrations, and involutions of ventral prostate and seminal vesicles	[50]
Rats	5, 10 and 20 mg/kg BW/day	Decreased sperm count and motility, increased abnormal sperm count, and reduced serum levels of testosterone, LH, and FSH	[47]
Bulls	16.4 mg/kg BW/day	Reduced sperm production and motility and increased proportion of sperm midpiece abnormalities	[51]
Bulls	8 mg/kg BW/day	Primary and secondary sperm abnormalities and increased number of sperm with proximal droplets	[52]

**Table 3:** Selected experimental studies describing effects of gossypol on female reproduction.

Animals	Gossypol dose	Effects	Reference
Rats	5 mg/kg BW/day	Longer diestrus	[53]
Rats	25 mg/kg/day	Lower levels of estradiol-17*β*	[54]
Rats	20 mg/kg/day	Irregular and longer estrous cycles, prolonged time for mating, decreased pregnancy rate, and reduced number of viable embryos	[55]
Heifers	~51 mg/kg BW/day	No interference on cycling, first service conception rate, and ovarian morphology	[56]
Heifers	5 g of free gossypol/animal/day	Reduced number of ovarian follicles >5 mm	[57]

**Table 4:** Preventive procedures for reducing gossypol toxicity.

Procedures	Reference
Heat treatment Roasting extrusion	[58] [58, 59]
Irradiation Gamma irradiation Electron beam irradiation	[156–158] [60, 61]
Fungal fermentation *Aspergillus niger* *Aspergillus oryzae* *Candida tropicalis* * Saccharomyces cerevisiae * * Geotrichum candidum *	[161–163] [62] [63–66] [63, 64] [67]
Nutritional supplementation Ferric sulfate*** *** *** *** ** **Sodium selenite Vitamin E	[1] [68] [69, 70]
